# Statistical analysis plan for a pragmatic phase III randomised controlled trial examining behaviour change physiotherapy intervention to increase physical activity following hip and knee replacements: the PEP-TALK trial

**DOI:** 10.1186/s13063-021-05362-x

**Published:** 2021-07-20

**Authors:** Alexander Ooms, Susan J. Dutton, Scott Parsons, Beth Fordham, Caroline Hing, Sarah Lamb, Toby Smith

**Affiliations:** 1 Oxford Clinical Trials Research Unit, Centre for Statistics in Medicine, Nuffield Department of Orthopaedics, Rheumatology and Musculoskeletal Sciences, University of Oxford, Oxford, UK; 2 Centre for Rehabilitation Research in Oxford, Nuffield Department of Orthopaedics, Rheumatology and Musculoskeletal Sciences, University of Oxford, Oxford, UK; 3 University of London St George’s Molecular and Clinical Sciences Research Institute, London, UK; 4 College of Medicine and Health Sciences, University of Exeter, Exeter, Devon UK; 5 Faculty of Medicine and Health Sciences, University of East Anglia, Norwich, UK

**Keywords:** Statistics, Randomised controlled trial, Joint replacement, Rehabilitation, Behaviour change

## Abstract

**Background:**

Total hip (THR) and total knee replacements (TKR) are two highly successful orthopaedic procedures that reduce pain for people with osteoarthritis. Previous evidence suggests that physical activity, at best, remains the same pre- to post-operatively, and in some instances declines. The PEP-TALK trial evaluates the effects of a group-based, behaviour change intervention on physical activity following a THR or TKR.

**Methods:**

PEP-TALK is an open, phase III, pragmatic, multi-centre, parallel, two-arm, two-way superiority randomised controlled trial investigating the effectiveness of usual care plus a behaviour change therapy compared with usual care alone following primary THR or TKR. The primary outcome is the UCLA Activity Score at 12 months post-randomisation which will be analysed using a linear mixed effects model. Secondary outcomes measured at 6 months and 12 months after randomisation include the UCLA Activity Score, Lower Extremity Functional Scale, Oxford Hip/Knee Score, Numerical Rating Scale for Pain, Generalised Self-Efficacy Scale, Tampa Scale for Kinesiophobia, Hospital Anxiety and Depression Scale, EuroQoL EQ-5D-5L index and EQ-VAS and complications or adverse events. Full details of the planned analysis approaches for the primary and secondary outcomes, as well as the planned sensitivity analyses to be undertaken due to the COVID-19 pandemic, are described here. The PEP-TALK study protocol has been published previously.

**Discussion:**

This paper provides details of the planned statistical analyses for the PEP-TALK trial. This is aimed to reduce the risk of outcome reporting bias and enhance transparency in reporting.

**Trial registration:**

International Standard Randomised Controlled Trials database, ISRCTN Number: 29770908. Registered on October 2018.

## Background

This analysis strategy adheres to the Statistical Analysis Plan Guidelines [1].

Total hip (THR) and total knee replacements (TKR) are two highly successful orthopaedic procedures which reduce pain for people with osteoarthritis [2, 3]. Over 230,000 THR and TKRs were performed in the UK in 2019 [2]. Approximately 90% of patients are satisfied following THR and TKR [3] with significant improvements in pain and physical function after 3 to 12 months [3, 4]. However, medical co-morbidities are common in this population. These include hypertension (56%) [5], cardiovascular disease (20%) [6], diabetes (16%) [6] and multi-joint pain (57%) [5]. Twenty-seven per cent of people who undergo joint replacement have three to four comorbidities [6]. Medical comorbidities such as these have a significant negative impact on both health-related quality of life and societal burden [7, 8].

Historically, it has been assumed that people are more active following THR or TKR through the amelioration of their joint pain [9]. However, previous evidence has indicated that physical activity, at best, remains the same from pre- to post-operatively, and in some instances declines [9]. There does not appear to be a difference in physical activity trajectories between those following THR or TKR [9, 10]. The reasons for reduced participation may differ between the groups [10] with TKR more often associated with increased pain in the initial 12 post-operative months compared to THR [3, 4], whereas those with THR may have greater fear avoidance through the risks of joint implant dislocation [3, 4].

Subsequent analyses from large USA and UK datasets have supported this finding, re-enforcing the notion that physical activity is lower after THR and TKR compared to age- and gender-matched cohorts who had not undergone joint replacement [10]. Given that physical activity can significantly reduce symptoms associated with common comorbidities [11], this population’s physical inactivity has a detrimental effect on their health. Participating in regular physical activity can decrease the risk of cardiovascular disease by 52% [12], diabetes by 65% [13] and some cancers by 40% [14] and reduces all-cause mortality by 33% and cardiovascular mortality by 35% [15]. Accordingly, supporting people to be more physically active can improve both patient’s health and decrease the economic burden these diseases place on the NHS. To date, no interventions aimed to increase physical activity following joint replacement surgery have been robustly tested. To address this, the PEP-TALK trial was undertaken.

## Methods

### Trial design

The trial is an open, phase III, pragmatic, parallel, two-arm, two-way superiority randomised controlled trial (RCT) on individuals investigating the effectiveness of usual care plus a group exercise and behaviour-change intervention versus usual care alone to increase physical activity following primary THR or TKR. Neither participants nor physiotherapists can be blinded to the treatment allocation. Primary comparison is assessed at 12 months post-randomisation with data being collected at baseline (pre-operatively), 6 months and 12 months post-randomisation.

Participants will be screened for inclusion in the trial pre-operatively, consented pre-operatively and eligibility confirmed post-operatively. They will then be randomised prior to hospital discharge and notified of their group allocation to facilitate the organisation of their rehabilitation. Initially, participants were randomised to the two groups 1:1 using minimisation by trial centre, type of joint replacement (THR or TKR), and Charlson co-morbidity index (1–3 or ≥4). The minimisation algorithm will have a probabilistic element of 0.8 included to ensure unpredictability of treatment assignment.

After 75 randomisations, the random allocation ratio was changed to 2:1 (Experimental Intervention: Usual Care). This change was made to ensure more participants were randomised to the experimental intervention group. The intervention is group-based and is designed to have three or more participants per group session. Based on evidence from early recruiting sites, there was difficulty to consistently fill sessions under a 1:1 allocation ratio. Therefore this change was deemed important to facilitate larger groups. This change was implemented by the Trial Management Group and approved by the Data Safety Monitoring Committee, Sponsor and Research Ethics Committee.

Those randomised to usual care (the comparator) will receive six, 30-min group-based exercise sessions. Those randomised to the experimental intervention will receive six group-based behaviour change intervention sessions (30-min duration) immediately followed by the control intervention of 30 min of group-based exercise and three follow-up telephone calls up to 6 weeks after completing the group sessions. Both group’s physiotherapy will commence within the initial four weeks post-randomisation and continue weekly for 6 weeks. Further details of the trial design and procedures, including full eligibility criteria and trial interventions are found in the PEP-TALK trial protocol [16].

### Outcomes

#### Primary outcome

The primary outcome measure is the UCLA Activity Score 12 months post-randomisation. The UCLA Scale is a reliable and valid self-reported tool to assess physical activity [17, 18] that assesses global activity levels with a grading system of 1 to 10 where 1 equates to “wholly inactive, dependant on others and cannot leave residence” and 10 refers to “regularly participates in impact sports”.

#### Secondary outcomes

The secondary outcome measures, self-reported with partial answers being coded as missing and collected at baseline (except complications), 6 and 12 months post-randomisation unless otherwise stated, are as follows:

- *Functional outcome:* Lower Extremity Functional Scale (LEFS) [19]. A questionnaire containing 20 questions scored using a scale 0-80 with a higher score representing a higher functional level.
- *Disease specific function:* Oxford Hip Score/Oxford Knee Score (OHS/OKS) [20, 21]. A 12-item disease-specific questionnaire, scores range from 0 to 48 where 48 indicates high joint function. Murray et al. [22] recommends to impute the mean value representing all other items to fill in two or fewer missing items.
- *Perceived level of pain*: Numerical Rating Scale (NRS) for Pain. An 11-point scale where participants mark their perceived pain between 0 representing ‘no pain’ to 10 representing the ‘worst possible pain’.
- *Self-efficacy:* Generalized Self-Efficacy Scale (GSES) [23]. A 10-item scale with scores ranging from 10 to 40, higher scores representing a high level of self-efficacy.
- *Fear avoidance:* Tampa Scale for Kinesiophobia [24]. A 17-item self-completed questionnaire with scores from 17 to 68 where the higher scores indicate an increasing degree of kinesiophobia.
- *Psychological distress:* Hospital Anxiety and Depression Scale (HADS) [25]. This scale consists of 14 items divided into two 7-item subscales: Anxiety and Depression. The total score is out of 42 (21 per subscale), higher scores indicate greater levels of anxiety/depression or global psychological distress.
- *Health-related quality of life:* Euroqol EQ-5D-5L [26]. A patient-reported health-related quality of life questionnaire consisting of two parts. First, five domains related to daily activities with a 5-level answer possibility are measured [26, 27], which will be converted into multi-attribute utility scores using established algorithm [28]. To calculate EQ-5D-5L Index scores the Crosswalk Index Value Calculator will be used to map the 5L descriptive system data onto the 3L dataset using the mapping function developed by van Hout et al. [27] as, at the time of writing this statistical analysis plan, there is still debate about the appropriate value set for the 5L. Secondly, the Euroqol VAS (EQ-VAS) is a 0 to 100 visual analogue score from the worse (0) to best health imaginable (100). Any participant who dies will have their EQ-5D-5L Index imputed as a score of zero for all time points after death, their EQ-VAS scores will be missing data for those time points.
- Complications and adverse events will be collected throughout the trial.

### Sample size

Two hundred fifty participants (125 per arm) are required to detect a standardised effect size of 0.4 with 80% power and 5% (2-sided) significance, allowing for 20% loss to follow-up. These calculations are based on the primary outcome at 12 months post-randomisation, assuming a baseline standard deviation of 2.5 [17] and a between-group difference of one. Our target standardised effect size is derived from the UCLA Activity Score’s minimal clinically important difference of 0.92 [17]. The sample size was increased to 260 to account for the change in allocation ratio, maintaining the same power and type I error rate.

### Effect of COVID-19 pandemic

The COVID-19 pandemic impacted on the conduct of the PEP-TALK trial. All elective surgeries, including THRs and TKRs, were cancelled as part of the UK national lockdown (23rd March 2020) and group-based physiotherapy classes within the hospital outpatient setting (a mechanism the trial relies on for both treatment groups) was stopped indefinitely.

A direct consequence of the cancellation of THRs and TKRs was that the trial was no longer able to randomise eligible and consented participants and was forced to close recruitment prematurely (230 final randomisations of the minimum sample size of 260).

As the trial had been open to recruitment for less than 12 months by March 2020, none of the randomised participants reached the full 12-month follow-up without being affected by the COVID-19 lockdown. This is particularly noteworthy in this instance as participants are likely to be in a demographic more medically vulnerable to the pandemic and all outcomes are assessed through Patient Reported Outcome Measures (PROMs). It is hypothesised the lockdown will be a confounder whilst assessing these outcomes, particularly those pertaining to more psychological aspects (e.g. the GSES) which may have been impacted by COVID-19 social measures on behaviour. However, as the trial is randomised, this effect should be the same across both treatment groups and therefore should not affect the overall treatment effect estimate.

An indirect consequence of the pandemic on the trial is that it is possible, in a ‘post-COVID-19 world’, that what is considered “usual care” will be different to how that was perceived at the time of the trial’s conception (2016–2017). This has a potential effect on the generalisability of the results. The trial is pragmatic by nature and every effort has been made to follow-up participants to ascertain what intervention they actually received. Through reporting this information, it is hoped this trial will give a non-conclusive indication of what usual care was during this change in practice as a result of the COVID-19 pandemic and the effectiveness of it.

Due to the effects of the pandemic, analysis and data exploration unforeseen when writing the protocol have been included in this analysis plan. These additions, found in the relevant section of this paper, will assess the effect of the pandemic on the trial and provide insights on future physiotherapy service configuration.

*Note:* The terms ‘COVID-19 status’ and ‘pre-COVID-19/COVID-19’ groups used within this paper refer to the definitions outlined in the ‘Definition of analysis populations’ section. This does not refer to participants who tested positive for COVID-19; testing information has not been collected as part of this trial.

### Statistical analysis

#### General analysis principles

There is one planned final analysis, which will occur 12 months after the final participant’s randomisation, allowing for an appropriate time for the data to be collected, cleaned and prepared for final analysis. There is no multiple testing as only a single primary outcome is considered. Significance levels used will be 0.05 and 95% confidence intervals will be reported. Any analyses not pre-specified will be exploratory in nature and a significance level of 0.01 will be used to declare statistical significance and 99% confidence intervals will be presented. No formal interim analysis or predefined early stopping rules are planned for this trial.

#### Definition of analysis populations

- *Intention-to-treat:* inclusion of all available randomised participants who will be analysed in the groups to which they were randomly allocated irrespective of non-compliance. If a participant has observed data on any of the follow-up time points, they will be included in the analysis.
- *Per protocol:* eligible participants who received the treatment they were randomised to with data on the primary outcome at 12 months. Participants who had major protocol violations/deviation (e.g. not have received the treatment they were allocated to) will be excluded from this population.
- *Strict compliers:* participants who fall under the Strict Compliance definition outlined in the ‘Compliance’ section.
- *Compliers:* participants who fall under the Compliance definition outlined in the ‘Compliance’ section.
- *Attenders:* participants who fall under the Attendance definition outlined in the ‘Compliance’ section.
- *Pre-COVID-19:* participants who completed their intervention before national UK lockdown (23rd March 2020) and had no disruption to their planned treatment.
- *COVID-19:* participants who did not receive any intervention before 23rd March 2020 or had their intervention delivery disrupted by the UK lockdown.

#### Descriptive analysis

**Fig. 1:**
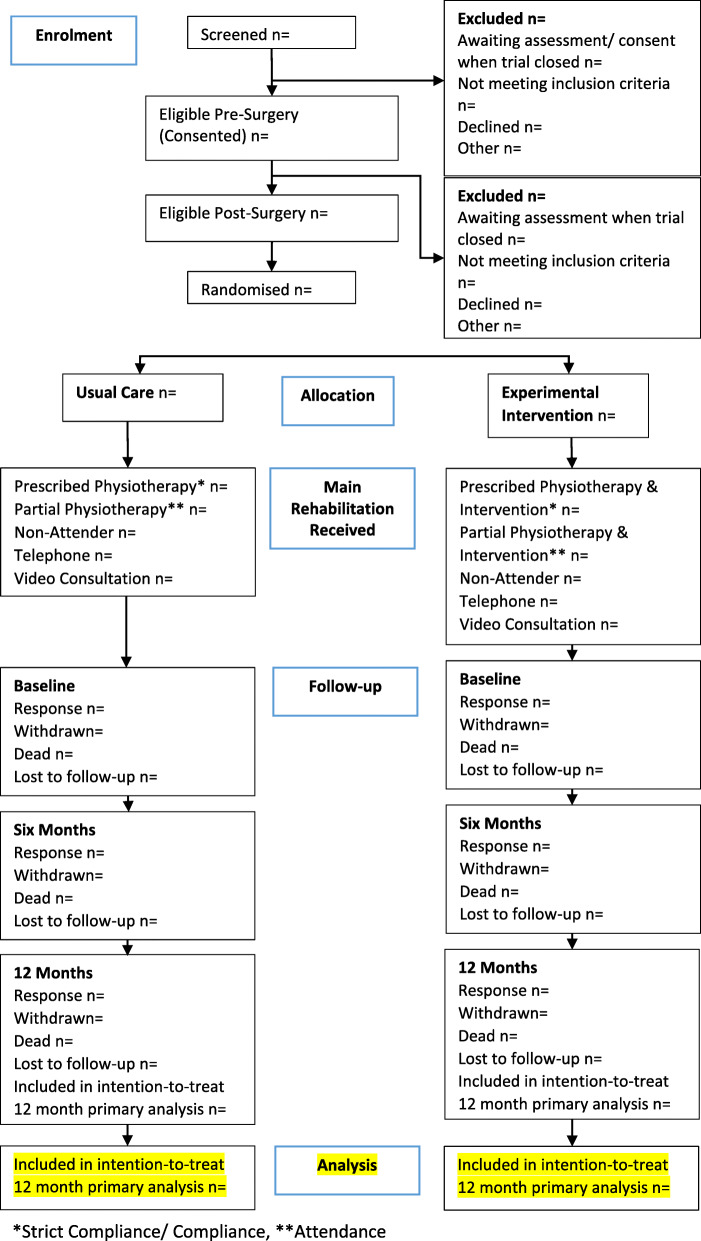
CONSORT diagram

The flow of participants through each stage of the trial, including the number of individuals screened, eligible, randomised to each group, receiving allocated treatment, and included in the primary analysis will be summarised using a CONSORT flow chart (Fig. 1). Reasons for ineligibility, loss to follow-up and exclusion from the primary analysis will be summarised. Participant follow-up data will be presented by randomised group as well as COVID-19 status (pre-COVID-19/COVID-19 as in the ‘Definition of analysis populations’ section).

Baseline characteristics will be reported by the treatment group, including the minimisation factors and important prognostic, demographic and clinical covariates. Numbers (with percentages) for binary and categorical variables and means (and standard deviations), or medians (with lower and upper quartiles) for continuous variables will be presented, there will be no tests of statistical significance nor confidence intervals for differences between randomised groups on any baseline variable. Baseline characteristics will also be reported by COVID-19 status in order to explore the difference in demographics between these groups.

It is likely that some data may not be available due to voluntary withdrawal of participants, lack of completion of individual data items or general loss to follow-up. The number (with percentage) of withdrawals from the trial and the numbers lost to follow-up for the primary outcome together with the associated reasons (where possible) will be reported by the treatment group. Any deaths (and their causes) will be reported separately.

#### Compliance

Deviations from intended treatment (non-adherence to the protocol) will be summarised for the randomised groups; these will include non-compliance and withdrawal of consent. Details of compliance and what intervention was actually received will be reported by the treatment group and also separately by COVID-19 status. Three levels of compliance: Strict Compliance, Compliance and Attendance have been defined as follows:

1. Strict Compliance (as defined in the original Protocol [16]):

*Usual Care group:*

- Attends at least four out of six physiotherapy sessions

*Experimental Intervention group:*

- Attends at least four out of six group intervention sessions with a minimum of three participants per session
- Receives two out of three follow-up telephone calls

2. Compliance:

*Usual Care group:*

- Attends at least four out of six physiotherapy sessions

*Experimental Intervention group:*

- Attends at least four out of six group intervention sessions with a minimum of three participants per session

3. Attendance:

*Usual Care group:*

- Attends at least one out of six physiotherapy sessions

*Experimental Intervention group:*

- Attends at least four out of six group intervention sessions.

Other indicators of compliance to the rehabilitation exercises (i.e. data collected from Exercise Diaries) may be summarised by treatment group in tabular or graphical form. The effect of changing the randomisation ratio from 1:1 to 2:1 after 75 randomisations on levels of compliance will also be explored.

#### Analysis of the primary outcome

The primary outcome measure, the role of usual care versus usual care plus the experimental intervention upon the UCLA Activity Score at 12 months post-randomisation, will be modelled using a mixed effects model. This model will account for person within centre random effects, and Charlson Comorbidity Index score and baseline UCLA Activity Score (as continuous outcomes), type of operation the patient is undergoing (THR or TKR), time (6 or 12 months) and treatment as fixed effects. Treatment by time point interactions will also be included in the model to allow time-specific treatment effects to be calculated. This model uses all available data at each time point. Comparison will be performed on an intention-to-treat basis and results presented as comparative summary statistics (i.e. difference in means) with 95% confidence intervals.

The appropriateness of the assumption of approximate normality of the residuals of this model will be assessed graphically. If the residuals are not normally distributed, the outcome data will be log-transformed to gain normality and geometric means with 95% confidence intervals will be reported. If data is not normally distributed after log-transformation, the non-parametric Mann-Whitney test will be used with no adjustment for baseline or stratification factors, and the difference in medians with 95% confidence intervals will be reported.

#### Supporting analyses of the primary analysis

An area under the curve (AUC) analysis will be performed for the UCLA Activity Score. Estimates will come from the same mixed model used in the analysis of the primary outcome except including baseline UCLA Activity Score in the ‘time’ fixed effect allowing time point-specific treatment effects to be calculated for baseline, six months and 12 months. These estimates will be used to calculate the AUC. Using the estimates from the mixed-effects model rather than raw, unadjusted estimates results in less bias estimates of the AUC when missing data are present [29].

Complier average causal effect (CACE) analyses will be performed to find estimates for the causal effect of actually receiving the treatment and the overall treatment effect (including non-compliers) through intention to treat analysis. The definitions of Strict Compliance, Compliance and Attendance will be used to perform three CACE analyses.

A supporting analysis of the primary outcome will use a three-level model with participants within predominant treating physiotherapist within the centre to examine the potential physiotherapist (random) effects. This model will formally incorporate terms that allow for possible heterogeneity in responses for participants due to the recruiting centre and the physiotherapist. The model will include the same fixed effects used in the primary analysis model as well as treatment by time point interactions.

An additional supporting analysis of the primary outcome using a reduced version of the primary analysis model, only using a person as a random effect, will be performed. This model is pertinent as Usual Care should be homogenous across the recruiting centres in a pragmatic trial so using a simpler model may yield a better-fit model. Model fit compared to the primary analysis model will be assessed using information criteria.

#### Analysis of the secondary outcomes

The continuous secondary outcomes, to compare functional outcomes, disease-specific function, perceived level of pain, self-efficacy, fear avoidance, psychological distress and health-related quality of life between groups, are assessed through the corresponding PROMs measured at baseline, 6 months and 12 months post randomisation. Mixed effects models, as used in the primary analysis, will be used to assess these outcomes. These models will account for person within centre random effects, and Charlson Comorbidity Index score and the relevant baseline PROM score (as continuous outcomes), operation type, time (6 or 12 months) and treatment as fixed effects. Treatment by time point interactions will also be included in the model to allow time-specific treatment effects to be calculated.

There is expected to be a low number of complications/serious adverse events (SAEs) in this trial. Any adverse events (AEs) occurring whilst a participant is continuing in the study, until completion of the final study visit will be recorded. All AEs will be reported and tabulated by grade and treatment group—similar reporting will be done with SAEs. The number of SAEs and the number of participants reporting one or more SAEs will be reported by the treatment group. If there is a large enough number of events for a comparison to be appropriate, then the complications in each group will be pooled and the ‘Total Complications’ analysed by calculating the odds ratio and 95% confidence interval using logistic regression adjusting for minimisation factors (recruiting centre, Charlson Comorbidity Index (as a continuous value) and type of operation) and treatment.

#### Sensitivity analyses

Sensitivity analysis will assess the internal validity of the trial results by performing a per-protocol analysis on all participants who fall under the per-protocol definition as per the ‘Definition of analysis populations’ section.

##### Missing data

Missing data analysis will be performed on the primary outcome only. The primary analysis multi-level model using repeated measures is relatively robust to missing data under the missing at random (MAR) assumption [30].

Analysis will be performed on an intention-to-treat basis. The number and percentage of participants in the missing category will be presented, as well as reasons for missingness if known. Missing data will be reported and summarised by the treatment group. The distribution of missing data will be explored to assess the assumption of data being missing at random under which the principal analyses will be conducted. Varying scores of the UCLA Activity Score (e.g. 30th, 40th, 50th, 60th, 70th quantiles) will be imputed where data is missing and these ‘complete’ datasets will be reanalysed, using the same model used in the primary analysis and the results presented in graphical form. This analysis will be undertaken if there is more than 5% missing data for the primary outcome at 12 months.

If there is evidence that there is a departure from the MAR assumption, a search for factors not included in the primary analysis model that explains missingness will be performed and if variables are found, multiple-imputation using chained equations [31] will be utilised, using the primary analysis model but including these variables to assess the sensitivity of the findings to missing data. If no variables are identified, multiple-imputation will not be performed.

#### Pre-specified subgroup analysis

All subgroup analyses will be on the primary outcome only. Subgroup analyses of the two clinical stratifying variables (type of operation and (THR or TKR), Charlson Comorbidity Index Score (1–3 or ≥ 4)) are planned. A subgroup analysis of COVID-19 status will also be performed. These will use an extended primary analysis model including an interaction term between treatment and each stratifying variable/COVID-19 status to define the subgroups. Subgroup analyses will be labelled as exploratory, and results will be interpreted with due caution, in line with recommendations for subgroup analysis made elsewhere [32]. The results will be presented in a forest plot.

#### Additional analysis

A mediation analysis will be carried out. A priori mediation analysis mediators will include self-efficacy, fear avoidance, pain and psychological distress to compare the mediation pathways presented in the BeST intervention [33] to the PEP-TALK intervention.

An additional analysis will be performed to assess the effect of COVID-19 on the activity at 12 months post-randomisation. The model used for the primary analysis will be extended to include COVID-19 status (as a fixed effect) and a COVID-19 status by time point interaction. The adjusted mean difference of COVID-19 status will be reported with supporting 95% confidence intervals. It should be noted that formally investigating COVID-19 status’ effect on activity is outside the scope of the original trial design so results from this analysis are hypothesis-generating and exploratory.

Descriptive statistics on secondary outcomes of GSES, the Tampa Scale for Kinesiophobia, HADS, EQ-5D-5L Index, EQ-VAS and NRS for pain may be produced to further assess the impact of COVID-19. No formal analysis to examine the relationship between COVID-19 status and secondary outcomes will be performed.

### Statistical packages

All analysis will be carried out using STATA [34] or R [35] statistical software. The package and version number used for analysis will be recorded and reported.

## Discussion

This paper provides details of the planned statistical analyses for the PEP-TALK trial to reduce the risks of reporting bias [36] and includes pre-specified analyses planned to explore the effect of COVID-19. Any changes or deviations from the analysis outlined in this paper will be described and justified fully in the final report.

## Trial status

The first participant was randomised into the study on the 12th of April 2019, final randomisation occurred on the 27th of March 2020. Randomisations were stopped due to COVID-19, 44 potential participants had consented and were awaiting surgery prior to randomisation when the trial closed. In total 230 participants, from eight participating centres, were randomised. Follow-up is currently ongoing and is expected to finish in April/May 2021 with the final data lock occurring in Summer 2021. All analyses being conducted thereafter.

## Data Availability

Not applicable.

## Abbreviations

AE: Adverse event
AUC: Area under the curve
CACE: Complier average causal effect
GSES: Generalized Self-Efficacy Scale
HADS: Hospital Anxiety and Depression Scale
LEFS: Lower Extremity Functional Scale
MAR: Missing at random
NRS: Numeric Rating Scale
OHS: Oxford Hip Score
OKS: Oxford Knee Score
PROM: Patient Reported Outcome Measure
SAE: Serious adverse event
THR: Total hip replacement
TKR: Total knee replacement
UCLA: University of California, Los Angeles
VAS: Visual analogue scale
